# Supplementary material for: The Construction and Exploration of a Comprehensive MicroRNA Centered Regulatory Network in Foxtail Millet (Setaria italica L.)
Source: Front Plant Sci. 2022 May 6;13:848474. doi: 10.3389/fpls.2022.848474 (PMC9121102; doi:10.3389/fpls.2022.848474)
Supplement: Supplementary file 2 [file Data_Sheet_2.zip › Sit-miR168_Seita.1G378700.1_527_TPlot.pdf]

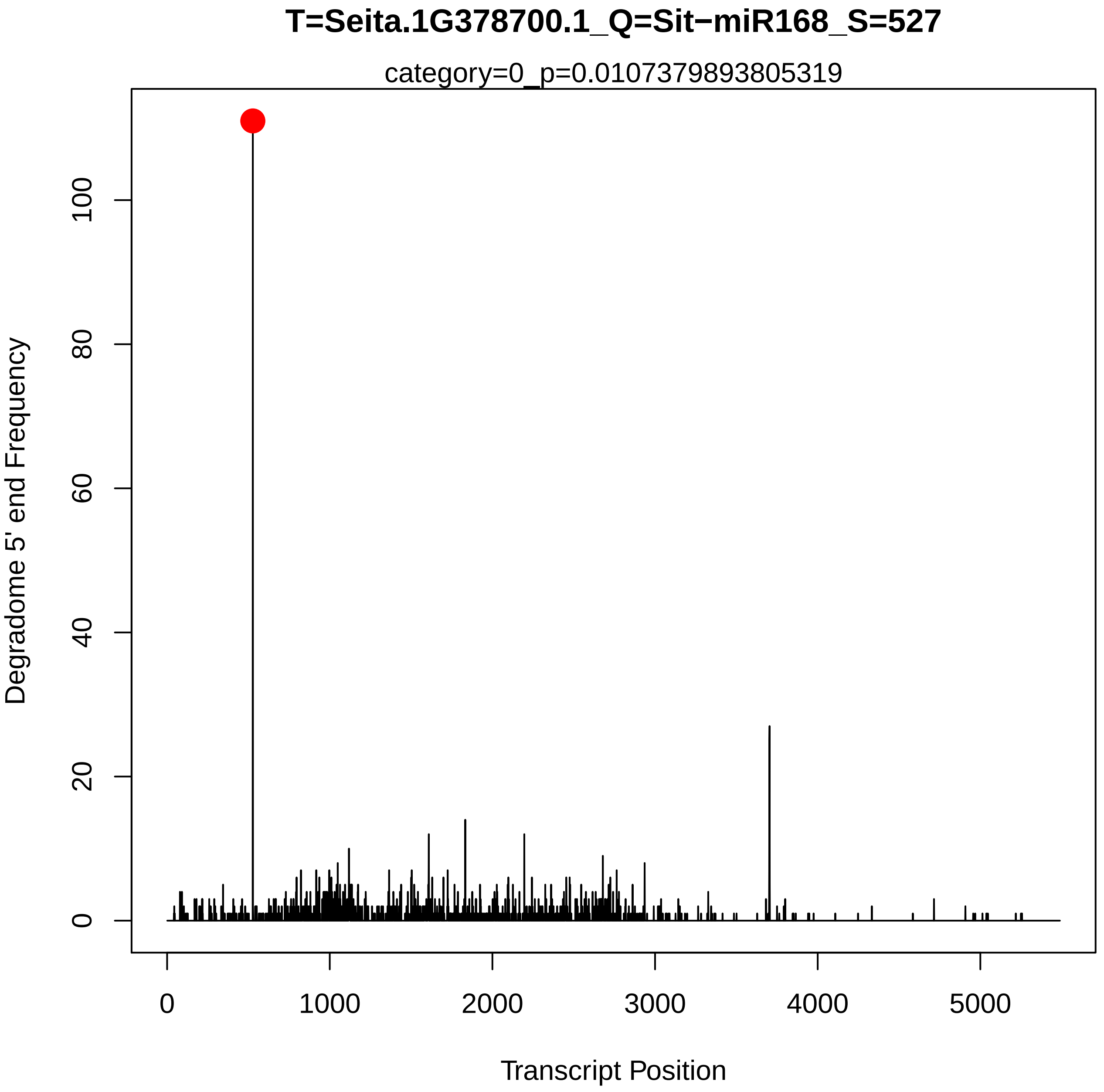

T=Seita.1G378700.1_Q=Sit−miR168_S=527
category=0_p=0.0107379893805319
Degradome 5' end Frequency
Transcript Position
0
20
40
60
80
100
0
1000
2000
3000
4000
5000
